# AATF/Che-1—An RNA Binding Protein at the Nexus of DNA Damage Response and Ribosome Biogenesis

**DOI:** 10.3389/fonc.2020.00919

**Published:** 2020-06-10

**Authors:** Rainer W. J. Kaiser, Johanna Erber, Katja Höpker, Francesca Fabretti, Roman-Ulrich Müller

**Affiliations:** ^1^Medizinische Klinik und Poliklinik I, University Hospital Ludwig-Maximilian-University Munich, Munich, Germany; ^2^Department II of Internal Medicine and Center for Molecular Medicine Cologne, Faculty of Medicine and University Hospital of Cologne, University of Cologne, Cologne, Germany; ^3^Cologne Excellence Cluster on Cellular Stress Responses in Aging-Associated Diseases (CECAD), University of Cologne, Cologne, Germany; ^4^Department I of Internal Medicine and Center for Molecular Medicine Cologne, University of Cologne, Cologne, Germany; ^5^Department of Medicine II, School of Medicine, Technical University of Munich, University Hospital Rechts der Isar, Munich, Germany; ^6^Systems Biology of Ageing Cologne, University of Cologne, Cologne, Germany

**Keywords:** AATF/Che-1, DNA damage response, p53, ribosome biogenesis, rRNA processing

## Abstract

The DNA damage response (DDR) is a complex signaling network that is activated upon genotoxic stress. It determines cellular fate by either activating cell cycle arrest or initiating apoptosis and thereby ensures genomic stability. The Apoptosis Antagonizing Transcription Factor (AATF/Che-1), an RNA polymerase II-interacting transcription factor and known downstream target of major DDR kinases, affects DDR signaling by inhibiting p53-mediated transcription of pro-apoptotic genes and promoting cell cycle arrest through various pathways instead. Specifically, AATF was shown to inhibit p53 expression at the transcriptional level and repress its pro-apoptotic activity by direct binding to p53 protein and transactivation of anti-apoptotic genes. Solid and hematological tumors of various organs exploit this function by overexpressing AATF. Both copy number gains and high expression levels of AATF were associated with worse prognosis or relapse of malignant tumors. Recently, a number of studies have enabled insights into the molecular mechanisms by which AATF affects both DDR and proliferation. AATF was found to directly localize to sites of DNA damage upon laser ablation and interact with DNA repair proteins. In addition, depletion of AATF resulted in increased DNA damage and decrease of both proliferative activity and genotoxic tolerance. Interestingly, considering the role of ribosomal stress in the regulation of p53, more recent work established AATF as ribosomal RNA binding protein and enabled insights into its role as an important factor for rRNA processing and ribosome biogenesis. This Mini Review summarizes recent findings on AATF and its important role in the DDR, malignancy, and ribosome biogenesis.

## AATF/Che-1—a Potent Modulator of the DNA Damage Response

Upon genotoxic stress, cells activate a complex network of signaling cascades in order to promote cell cycle arrest allowing for DNA repair or programmed cell death. This signaling network is known as the DNA damage response (DDR) and contains some of the most important molecular players that mediate genomic stability (1–5). One of these players is the tumor suppressor protein p53, a well-known anti-proliferative transcription factor and key target of mutations in ~50% of human malignancies (6). Following DNA damage, p53 is phosphorylated and thereby stabilized by upstream kinases such as ATM/ATR and CHK1/CHK2 (1, 4). In response, p53 promotes the transactivation of either cell cycle-arrest (e.g., CDKN1A, RPRM, GADD45) or pro-apoptotic genes such as PUMA, BAX, or BAK (4). Cell cycle arrest allows for the essential time to repair DNA damage—a pre-requisite for cellular physiology and a pivotal step to prevent proliferation of cells that propagate a damaged genome. Ensuring cellular survival through cell cycle arrest and repair may be considered the primary aim after structural damage to DNA. However, in the case of excessive damage that goes beyond any possibility of repair, cells undergo apoptosis or senescence to avoid genome instability and malignant transformation. By supporting both mechanisms, p53 prevents uncontrolled proliferation (3, 7, 8).

**Figure 1:**
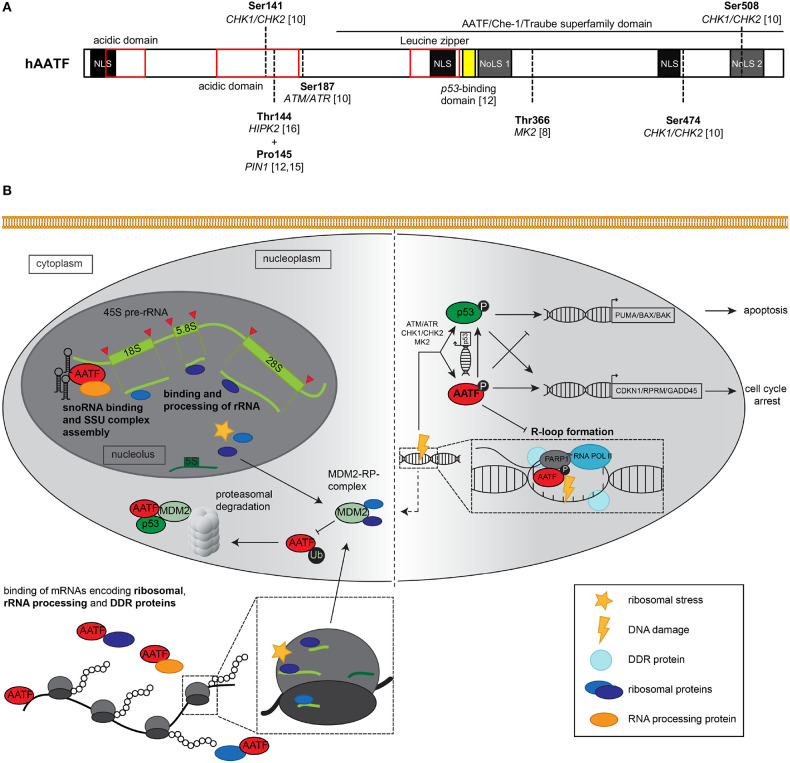
**(A)** Schematic representation of the AATF/Che-1 protein structure. The 560 amino acids containing protein consists of three nuclear localization sites (NLS), two nucleolar localization sites (NoLS, dark gray) two N-terminal acidic domains (red), and a leucin zipper as classical DNA binding domain (red). Multiple serine and tyrosine residues may be phosphorylated by kinases such as CHK1/CHK2, ATM/ATR as well as MK2 and HIPK2 (8, 10, 11); in addition, proline 145 is isomerized by prolyl isomerase 1 (PIN1) (12, 13). The C-terminal part of the protein displays great homology to the murine homolog Aatf/Traube and is therefore known as AATF/Che-1/Traube superfamily domain. AA 305–323 (yellow) have been shown to be essential for the interaction of AATF with p53 protein (12). **(B)** Graphical summary of nucleolar, nucleoplasmic, and cytoplasmic functions exerted by AATF and its response to ribosomal stress and DNA damage. Left panel: Nucleolar AATF is involved in binding and processing of ribosomal RNA, e.g., through binding of both 45S pre-rRNA (light green) and snoRNA (black hairpins) along with other RNA binding proteins that may process rRNA. In the nucleus and cytoplasm, AATF binds to mRNA encoding ribosomal proteins, rRNA processing enzymes as well as proteins involved in the DDR, which may the respective proteins' expression levels. Upon ribosomal stress, RPs (dark blue, purple) from the nucleolus as well as RPs from disassembled ribosomes shuttle to the nucleoplasm, where they may interact with E3 ubiquitin ligase MDM2. MDM2 is able to ubiquitinate AATF after isomerization by PIN1 (not shown), which subsequently leads to proteasomal degradation of AATF. In addition, AATF directly binds both p53 and MDM2 protein. Right panel: Upon DNA damage, canonical DDR kinases such as ATM/ATR, CHK1/CHK2, and MK2 phosphorylate and thereby active AATF [(for specific phosphorylation sites, see panel **(A)**], which in turn impacts on p53 transcriptional activity and may itself transactivate anti-apoptotic genes such as CDKN1 or RPRM. In addition, AATF relocates to sites of DNA damage, where it interacts with DNA repair protein PARP1 and may play a role in DNA repair. Recently, AATF was shown to inhibit R-loop formation, while knockdown of AATF led to increased frequency of R-loops and DNA damage. Excessive genotoxic stress may also activate MDM2 (dashed arrow), leading to proteasomal decay of AATF and transactivation of pro-apoptotic genes by p53. ATM, Ataxia Telangiectasia Mutated serine-protein kinase; ATR, Ataxia telangiectasia, and Rad3 related serine-protein kinase; BAK, Bcl-2 homologous antagonist/killer; BAX, Bcl-2-associated X protein; CHK1/2, Serine/threonine-protein kinase Chk1/2; CDKN1, Cyclin dependent kinase inhibitor 1; GADD45, Growth arrest and DNA damage-inducible protein GADD45 alpha; MDM2, Mouse double minute 2 homolog; MK2, MAP kinase-activated protein kinase 2; PARP1, Poly(ADP-ribose) polymerase 1; PUMA, p53 upregulated modulator of apoptosis; RNAPII/RNA POL II, RNA polymerase II; RPRM, Protein reprimo.

Even though many details that dictate the choice between cell cycle arrest and apoptosis remain incompletely understood, a number of different factors influencing the role of p53 regarding these two opposing outcomes have been identified in recent years. One of these factors is the Apoptosis Antagonizing Transcription Factor (AATF)/Che-1, an RNA-polymerase II-interacting transcription factor (9). In 2006, Bruno et al. (10) published the first line of evidence pointing toward a close interplay of p53 and AATF and described that the upstream DDR kinases ATM/ATR and CHK2 can phosphorylate AATF at specific residues following genotoxic stress (Figure 1A). Phosphorylation stabilized the protein and was shown to trigger p53 and p21 gene expression (10, 14).

Indeed, AATF interacts with p53 on multiple levels. A study from our group (8) showed that DNA damage leads to phosphorylation of AATF residue T366 by another DDR kinase, the MAP kinase-activated protein kinase 2 (MK2), resulting in dissociation of a cytoplasmic complex consisting of AATF and myosin regulatory light chain 3 (MRLC3). Release from MRLC3 leads to the nuclear translocation of AATF. In the nucleus, AATF inhibits the p53-driven activation of pro-apoptotic genes such as BAK, BAX, and PUMA by binding their promoters. This effectively leads to cell survival by favoring cell cycle arrest over apoptosis (Figure 1B) (7, 8). Besides its effects on these downstream events, AATF also influences the abundance of p53 by increasing its basal transcription rate through direct binding to its promoter (12, 15, 16). In addition, Desantis et al. (12) established that AATF directly binds p53 protein through interaction of the DNA binding domain of p53 (amino acids 251–293) and amino acids 305–323 of AATF (Figure 1A). Following moderate DNA damage, AATF, p53, and BRCA1 form a ternary complex in the nucleus, which is able to activate cell cycle arrest genes. However, upon exceeding a certain threshold of DNA damage, the prolyl isomerase PIN1 isomerizes AATF, which leads to phosphorylation by Homeodomain-interacting protein kinase 2 (HIPK2) and subsequent ubiquitination of AATF by nucleoplasmic MDM2, dissociation of the ternary complex and transactivation of pro-apoptotic genes by p53 (11–13).

In a recent study (17), our group focused on the role of AATF in *KRAS*-driven lung adenocarcinoma and its connection to p53 and the DDR. Mechanistically, Welcker et al. (17) described that AATF suppressed TP53-induced transcription of pro-apoptotic genes, favored cell cycle arrest over apoptosis, and thereby contributed to proliferation and tumor progress of lung adenocarcinoma. In line with these findings, depletion of AATF prevented tumor progression in a p53-dependent manner. Interestingly, the inducible knockout of AATF in existing carcinomas halted tumor growth *in vivo* and significantly increased the overall survival rate in a mouse model of adenovirus-induced lung adenocarcinoma (17).

## Novel Aspects of the Interplay of AATF and the DDR

More recently, another study from our group described new findings regarding the interaction between AATF and the DDR. Jain et al. (18) showed that depletion of AATF led to an increase in DNA damage in murine kidney cells. Besides, loss of AATF in a mouse model resulted in a decrease in proliferative activity, an increase in apoptosis and reduced tolerance toward genotoxic stress. Of note, cells lacking AATF exhibited an increase in DNA double strand breaks both *in vitro* and *in vivo* as assessed by comet assays and immunohistochemical analyses. Mechanistically, we found that AATF depletion resulted in an increase in R-loops, transcription intermediates consisting of RNA:DNA hybrids, and displaced single strand DNA. In the setting of increased DNA damage and replicative stress, these R-loops can lead to DNA double strand breaks (DSB), and provide a possible explanation for the increase in DSB and thus genomic instability (19). Supporting a direct involvement of AATF at the site of DSB, AATF localized to the site of DNA damage upon laser ablation of cells expressing a GFP-tagged AATF fusion protein. In addition, AATF co-localized with the known DNA repair protein PARP1 at sites of DNA damage. Thus, AATF is a novel player in R-loop formation (18). This finding is supported by a recent study by Cristini et al. (20) that established AATF as part of the R-loop protein interactome. Interestingly, loss of AATF in the distal nephron resembled a clinical and histopathological phenotype of nephronophthisis (NPH) in our mouse model (18). NPH is an early-onset cystic kidney disease characterized by hypotrophic kidneys and progressing fibrosis. Importantly, DNA-damage signaling had been hypothesized before to be involved in the pathogenesis of this disorder (21, 22).

Both studies from our group again underline the close interplay between p53, the DDR, and AATF as well as the clinical significance of this complex connection. At the nexus of DNA repair and proliferation, AATF could serve as a pharmacological target for novel antineoplastic therapies. In addition, the close resemblance of the proximal tubule AATF knockout with human nephronophthisis suggests the DDR as a potential therapeutic target in order to extend the currently limited therapeutic options for NPH patients and halt the progression to chronic kidney disease.

## AATF, Nucleoli, and the Ribosome—the Key to Pro-Proliferative Activity of AATF and an Explanation to Embryonic Lethality of Its Knockout?

In spite of these promising therapeutic aspects, targeting AATF in a therapeutic fashion remains challenging, since complete ablation of AATF protein function leads to embryonic death as well as severe proliferation defects in the targeted tissue. Of note, AATF^−/−^ embryos showed a reduced number of total cells as well as growth arrest at the morula stage (embryonic day E3) with consecutive cell death (17, 23). In line with these results, we found the keratinocyte-specific knockout of AATF to result in severe skin proliferation defects, a completely disrupted skin barrier, and subsequent postnatal death (17). An explanation to this phenotype may be found in an early study by Thomas et al. (23), who first described and characterized the murine homolog of AATF *in vivo*. To explain the embryonic lethality of loss of AATF, the authors performed ultrastructural imaging studies of whole-body knockout mouse embryos, which revealed a decrease in ribosome and polysome density as well as rough endoplasmatic reticulum in response to loss of AATF. With ribosomes, the molecular machinery essential for protein synthesis (24, 25), being key players at the nexus of anabolism and cell growth, these findings suggested a possible function of AATF/Traube in ribosomal biogenesis for the first time. In spite of this interesting finding—considering the importance of the ribosome for cellular proliferation (26, 27)—it took nearly two decades for the scientific community to explore this function of AATF in more detail. Besides, ribosome biogenesis is also clearly linked to p53 activation—with ribosomal stress inhibiting the MDM2-mediated degradation of p53(27)—making a function of AATF in this process a highly intriguing topic. Recent imaging studies (28, 29) showed a primary localization of AATF to the nucleolus, the site of rDNA transcription, rRNA modification, and ribosome assembly (30). The first functional link between AATF and ribosome biogenesis came from two siRNA-based screens in which AATF was identified as critical co-factor of both 40S ribosomal subunit assembly and rRNA processing (29, 31). In parallel to these screening approaches in human cells, the yeast homolog of AATF, Brefeldin A resistance protein 2 (BFR2), was shown to participate in rRNA processing and RNA splicing as part of the yeast small subunit (SSU) processome (32). Additional hints to the close interplay of AATF and ribosomes included its detection in a number of ribosomal protein interactomes as well as nucleolar proteomes (33–35).

More recently, Bammert et al. (36) revealed that AATF formed an RNase- and salt-stable complex with the nucleolar proteins NGDN and NOL10, termed the AATF-NGDN-NOL10 (ANN) complex. Depletion of either component of this complex resulted in processing defects of rRNA, namely the accumulation of 30S pre-rRNA and depletion of 18S rRNA, as well as defects in nucleolar 40S ribosomal subunit assembly. However, the molecular mechanism underlying these defects remained unclear. Specifically, none of the three ANN proteins was shown to bind RNA directly, nor were RNA binding domains identified for any of the three.

Interestingly, in parallel to these studies, we and others (37–41) had identified AATF as an RNA-binding protein using RNA interactome capture (39, 40). Based on this finding, our group validated the RNA-binding capacity of AATF (42). Using independent techniques—enhanced Crosslinking and Immunoprecipitation (eCLIP) (43) as well as RNA co-immunoprecipitation followed by RT-qPCR (RIP-qPCR)—RNA targets bound by AATF and their respective binding sites were identified. Strikingly, the majority of bound transcripts consisted of ribosomal RNA, with the pre-ribosomal 45S RNA being one of the most enriched transcripts. Of note, AATF-bound ribosomal RNA far exceeded the level of input rRNA, which is the most abundant RNA biotype at baseline. This finding was shown to be specific to AATF when comparing the eCLIP enrichment of both nucleolar and non-nucleolar RBPs included in the ENCODE database. Interestingly, the binding sites of AATF in 45S pre-rRNA are localized in close proximity to the cleavage sites for rRNA maturation (42). Bammert et al. (36) had already established that depletion of AATF led to an accumulation of early rRNA precursors, and our data showed that this was accompanied by a decrease in 18S rRNA. Of note, AATF did not only bind to rRNA but was also associated with other RNA species required for ribosome biogenesis such as snoRNAs—essential players in rRNA maturation (44).

To further investigate the link between ribosome biology and AATF, we identified the protein interactome of AATF by affinity-purification mass spectrometry (AP-MS). Out of 160 bona-fide interactors a large proportion (almost 80%) turned out to be RNA binding proteins themselves, with 15% being ribosomal proteins (42). The majority of these interactions appeared to be mediated by direct protein-protein binding and was not affected by nuclease treatment. The combination of the protein and RNA interactome data indicates AATF to be a central hub in several small subunit (SSU) processing complexes. These complexes, consisting of ribosomal and nucleolar proteins as well as several RNA biotypes including snoRNA, are essential for both rRNA transcription and processing, crucially affecting ribosome assembly (44, 45).

These data are in line with evidence recently published by Pineiro et al. (46), who identified the RNA-polymerase I (RNAPI)-transcribed RNA-protein interactome and established AATF as an RNAPI-associated RBP itself. Again, the authors were able to show the specific binding of AATF to rRNA-precursors, namely 45S pre-rRNA (42).

With nucleoli being the prime location for ribosome synthesis and therefore crucially contributing to cell metabolism and proliferation (30), the nucleolar localization of AATF is highly interesting for a number of reasons. On the one hand, the decreased proliferation rate observed in full body and conditional knockouts of AATF could be explained by a loss of mature ribosomal RNA. On the other hand, AATF could be part of the nucleolar response to a variety of stressors, namely anti-proliferative stimuli and proteostatic imbalance, or even nucleolus-specific reactions to DNA damage leading to p53 activation.

Such nucleolar-specific reactions upon DNA damage or disrupted ribosome biogenesis are known as “nucleolar or ribosomal stress” (47, 48). Indeed, many ribosomal proteins influence p53-signaling by binding and deactivating the nucleoplasmic ubiquitin ligase MDM2, thereby stabilizing and activating p53, leading to either cell cycle arrest or apoptosis. This signaling cascade, which can be activated by ribosomal proteins shuttling from both nucleolus and cytoplasm into the nucleoplasm, is known as the RP-MDM2-p53 pathway (27, 49). Vice-versa, MDM2 can inhibit several ribosomal proteins through ubiquitination upon sensing defects in ribosome biogenesis, as was shown for the large ribosomal subunit protein RPL26. Reduction of cytoplasmic RPL26 levels led to destabilization of p53 mRNA with subsequent decrease of p53 protein levels and prevention of apoptosis, allowing for restoration of ribosome homeostasis rather than immediate cell death (50). AATF, which was shown to physically interact with both ribosomal proteins and MDM2 (13, 36, 42) and which is known to localize to nucleoli, the nucleoplasm, and the cytoplasm (8, 23, 28, 42), could also act as an essential effector and influence signaling outcomes of the RP-MDM2-p53 pathway throughout cellular compartments. The exact means, by which AATF influences the interplay of RP stress responses and MDM2 as well as its impact on p53 in the setting of nucleolar stress, remain to be investigated.

In summary, AATF joins a group of proteins that are essential for ribosome biogenesis, including pivotal steps of rRNA processing and nucleolar integrity, but which also fulfill numerous extra-nucleolar functions.

## Links Between the Molecular Function of AATF and Its Impact on Human Disease

The studies described above add to a list of publications that promote AATF as a central player in human diseases and an attractive therapeutic target. Indeed, AATF was shown to be an important oncogenic factor in both solid and hematological malignant diseases. In 2012, we could show that copy number gains at the *AATF* locus negatively correlated with overall survival in p53-proficient neuroblastoma patients (8). More recently, cell lines derived from neuroblastoma patients were shown to overexpress AATF (51). In addition, endometrial carcinoma samples exhibited a nuclear accumulation of AATF, suggesting activation of the AATF-mediated transcriptional program. Overexpression of a phospho-mimicking, hyperactive AATF mutant resulted in tumor resistance toward anti-neoplastic therapy (8). In a recent study, Desantis et al. (52) established that AATF expression levels positively correlated with progression and risk of relapse in multiple myeloma patients. Mechanistically, AATF expression led to an increase in stress-induced autophagy following mTORC1 inhibition, which promoted plasma cell survival *in vivo*. Kumar et al. (53) found that AATF promoted progression from non-alcoholic steatohepatitis to hepatocellular carcinoma by enhancing STAT3-mediated expression of monocyte chemoattractant protein 1 (MCP-1). In line with these findings regarding a pro-proliferative function of AATF in solid tumors (17), Folgiero et al. (54) described a similar effect of AATF in B-cell precursor acute lymphoblastic leukemia (BCP-ALL), where c-Myc driven AATF signaling promoted lymphoblast proliferation and ALL progression. Interestingly, the role of AATF in ribosome biology may contribute to the observed pro-prolifeative role of the protein in malignant disease, adding to the distinct mechanisms proposed above. Since rRNA transcription and ribosome biogenesis are required for cell growth and division, AATF-driven increase in ribosome density may contribute to the proliferative potential of malignant tumors. In line with this hypothesis, elevated ribosome synthesis has been shown to be a risk factor of malignancy (26), prompting the development of antineoplastic therapies that target the ribosome (55).

**Figure 2:**
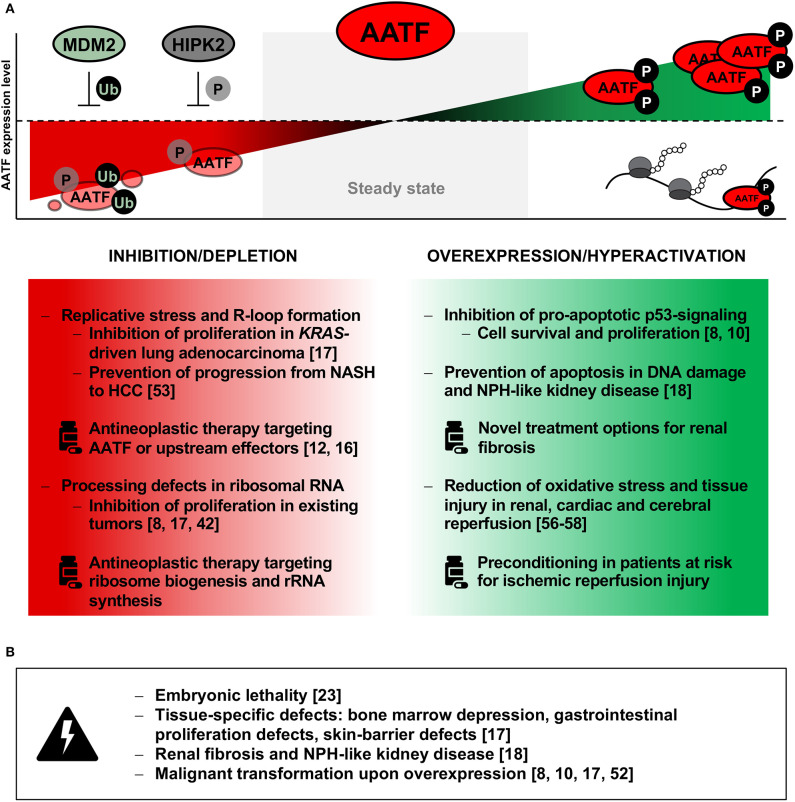
**(A)** Summary of observed effects upon inhibition or depletion (left panel) as well as overexpression or hyperactivity of AATF (right panel) and putative therapeutic approaches on modulating AATF. Possible treatment opportunities include degradation of AATF by targeting upstream inhibitors such as HIPK2 (phosphorylation) or MDM2 (ubiquitination), while hyperactivity of phosphorylated AATF may lead to increase in RNA binding and ribosome density with subsequent increase in proliferative activity. **(B)** Possible adverse events upon interference with AATF activity as deducted from available data are listed in the bottom panel. HCC, hepatocellular carcinoma; HIPK2, Homeodomain-interacting protein kinase 2; MDM2, mouse double-minute homolog 2; NASH, non-alcoholic steatosis hepatitis; NPH, nephronophthisis; P, phosphorylation; Ub, ubiquitination.

Aside from cancer, a number of studies also revealed putative roles of AATF in non-malignant, e.g., cardiovascular diseases. Models of ischemic reperfusion injury in both proximal tubular epithelial cells of the kidney and cardiomyocytes described an anti-apoptotic effect of AATF and could trace this effect to a reduced amount of reactive oxygen species (56, 57). More recently, Guo et al. (58) showed that AATF also protected neurons from oxygen and glucose deprivation-induced apoptosis, while loss of AATF resulted in aggravated neuron death *in vitro*. These findings suggest a potential role for activating AATF to mediate organ protection when exposed to damaging stimuli (Figure 2).

However, modulation and specifically inhibition of AATF is not likely to become a simple therapeutic option unless limited to a short period of time or specifically targeted to the tissue of choice. Indeed, AATF is expressed ubiquitously and is essential for the growth and homeostasis of all normal tissues examined to date. As mentioned above, AATF was shown to be indispensable for proliferation and cellular survival by Thomas et al. (23) more than 20 years ago. Since global depletion of AATF therefore does not appear to be promising, therapeutic options may involve a more subtle approach. This may include targeting essential domains of AATF that are involved in the binding to DNA and subsequent transcription factor activity, such as small-molecule inhibitors targeting the protein's leucine zipper domain—a mechanism already proven to be successful in the inhibition of transcription factor c-Myc (59). Pharmacological modulation of upstream interactors of AATF is another promising approach. For instance, increasing the activity of either MDM2 or HIPK2 may lead to enhanced ubiquitination and proteasomal decay of AATF (Figure 2A), while targeting another interactor of AATF, MRLC3, may result in cytoplasmic retention of both proteins, preventing both nuclear shuttling of AATF and the initiation of its anti-apoptotic transcriptional program (8, 11, 12).

Design of such specific inhibitors and modulators is usually preceded by another step—the ultrastructural characterization of the targeted protein, enabling the identification of vulnerable and druggable domains. Considering the novel role of AATF as RNA binding protein, interference with transcript-specific RNA binding events pose another therapeutic approach. The design and application of antisense oligonucleotides (ASO) targeting specific binding motifs have been shown to effectively reduce protein-RNA interactions and, for example, subsequent processing of RNA (43). Regarding AATF, ASOs designed to target its binding sites in 45S pre-rRNA, may lead to deficient rRNA synthesis, a halt in proliferation and other significant downstream effects (60). Whichever the therapeutic molecule of choice—broadening our knowledge of the spatiotemporal functions and characteristics of AATF in both healthy and pathological states will be pivotal to advancing therapeutic options (Figures 2A,B).

## Conclusion: RNA-Binding AATF Functions in Both Ribosome Biogenesis and DDR

Recent work has shed much light on the function of AATF and characterized it as a key protein both in ribosome biogenesis and DDR (Figure 1B). Whilst it is intriguing to speculate that both functions are closely related and depend on each other—considering the interplay between ribosomal stress and p53 via MDM2—this aspect will require future studies, including investigation of the exact nature of RNA-protein and protein-protein interactions exerted by AATF through unraveling its ultrastructure. Such studies will be of utmost importance to obtain a better understanding of the molecular role of AATF in cellular biology and human disease. Importantly, this knowledge will—in the long term—be the key to exploiting a potential modulation of AATF in the clinical setting with a focus on cancer, cardiovascular disease, and organ protection from genotoxic and oxidative stress.

## Author Contributions

RK wrote the manuscript and designed the figures. JE reviewed the manuscript and designed the figures. R-UM, KH, and FF reviewed and edited both manuscript and figures and finalized the manuscript. All authors read and approved the final version of the manuscript.

## Conflict of Interest

The authors declare that the research was conducted in the absence of any commercial or financial relationships that could be construed as a potential conflict of interest.
